# The Risk Factors for Undiagnosed and Known Hypertension among Malaysians

**DOI:** 10.21315/mjms2019.26.5.9

**Published:** 2019-11-04

**Authors:** Ooi Wei Lim, Chen Chen Yong

**Affiliations:** 1 Malaysia Foundation Programme, Heriot-Watt University Malaysia, Putrajaya, Malaysia; 2 Faculty of Economics and Administration University of Malaya, Kuala Lumpur, Malaysia

**Keywords:** undiagnosed hypertension, known hypertension, modifiable risk factors, non-modifiable risk factors

## Abstract

**Background:**

The prevalence of known hypertension has resulted from the progression of undiagnosed hypertension. This study is targeted to examine and compare the risk factors based on the estimated odds ratios of modifiable and non-modifiable risk factors on different outcome levels of hypertension.

**Methods:**

A nationwide representative secondary data from the Fourth National Health of Morbidity Survey (NHMS IV) which consists of 24,632 non-institutionalised Malaysian population conducted by the Ministry of Health in 2011 has been used. Odds ratio (OR) with 95% confidence interval has been estimated using multinomial logistic regression.

**Results:**

Obese and overweight respondents exhibit increased likelihood of having undiagnosed and known hypertension. Physically inactive, ex-smokers and unclassified drinkers are found having higher likelihood to have known hypertension. However, current drinkers are found to have higher likelihood of having undiagnosed hypertension. Elderly, retirees, home makers and lower educated respondents are shown higher odds to have undiagnosed hypertension. Likewise, the likelihood of having known hypertension has been found to increase among the elderly and other Bumiputra.

**Conclusion:**

Through this research, significant predictors which consist of obese and overweight respondents, current drinkers, older respondents (above 65 years old) and primary educated respondents are having higher likelihood to have undiagnosed hypertension.

## Introduction

Hypertension has been identified as the leading risk factor for death, claiming 1.5 million lives each year in Malaysia (1). The number of individuals with hypertension in developing countries is estimated to be 1.17 billion, which represents almost three-fourths of the world’s hypertensive population (2). Although Malaysia began its Healthy Lifestyle Campaign back in 1991, no decrease in the prevalence of hypertension has been observed. For example, about RM215.9 million was spent on anti-hypertensive medicines alone in 2005 in Malaysia (3). Moreover, the prevalence of undiagnosed hypertension was 19.8% (95% CI: 19.0, 20.7), which was higher than that of known hypertension at 12.8% (95% CI: 12.2, 13.5) among adults above 18 years old (4).

The early identification of undiagnosed hypertension can help to prevent and reduce the progression of this disease, which may lead to serious complications including stroke and heart disease (5). Furthermore, the early detection of undiagnosed hypertension may prevent the increase in known hypertension, which may cause an alarming increase in the total prevalence of hypertension status in the country. Thus, investigating and estimating the odds ratios (OR) of modifiable and non-modifiable risk factors among individuals with undiagnosed hypertension in Malaysia is important to identify the potential predictors that can assist the government in the early detection of the prevalence of undiagnosed hypertension to prevent the occurrence of hypertension in the country.

Previous literature reviews show that the estimation of the predictors of hypertension outcomes is not only focused on the modifiable risk factors but also on the sociodemographic and socioeconomic factors (non-modifiable risk factors). Therefore, this study fills the gap in the literature by analysing and identifying the predictors (modifiable risk factors) at different levels of hypertension outcomes, that is, the one-to-one relationship among these factors. This work provides a detailed analysis to estimate the predictors (risk factors) of hypertension based on different hypertension outcomes, for example, undiagnosed and known hypertension. The objective of this study is to examine the odds of modifiable and non-modifiable risk factors at the different outcome levels of hypertension from different cultural backgrounds to provide insight for policy makers to develop the most cost-effective strategies for the prevention and control programmes of hypertension in Malaysia.

## Methods

### Data

This research used data from the Fourth National Health and Morbidity Survey 2011 (NHMS IV), which involved 24,632 eligible respondents. Using SPSS 23, the multinomial logit model was used to estimate the odds ratio (95% CI). The NHMS IV is a population-based study that included non-institutionalised individuals residing in Malaysia for at least two weeks prior to the data collection. Those who are staying in hotels, hostels and hospitals, among others, are excluded from this survey (4).

In the sampling frame of the NHMS IV, which was provided by the Department of Statistics Malaysia, Malaysia was divided into enumeration blocks (EBs) with geographically continuous areas. A total of 794 EBs were selected from the total EBs in Malaysia, and 484 and 310 EBs were randomly selected from urban and rural areas, respectively (4). Structured questionnaires with face-to-face interviews and the administered methods were used by the Ministry of Health, Malaysia, to collect data. This study was registered under the National Medical Research Registry (NMRR-12-324-11225).

### Variables

The outcome variable used in the present study is hypertension, which is identified as the dependent variable, and it has three outcome categories: ‘no hypertension’, ‘undiagnosed hypertension’, and ‘known hypertension’. Undiagnosed hypertension is defined as not known to have hypertension and as having an average systolic blood pressure equal to or more than 140 mmHg and/or diastolic blood pressure equal to or more than 90 mmHg. Known hypertension is defined as self-reported by the subject and having previously been diagnosed with hypertension by medical personnel. No hypertension is defined as individuals with no hypertension (4). In accordance with the NHMS IV, the blood pressure check-up was performed by nurses. The validated and calibrated Omron Japan Model HM-907 was used for blood pressure assessment.

All of the independent variables were categorical. The modifiable risk factors included physical activity (inactive and active), fruit and vegetable consumption (inadequate and adequate), drinking status (unclassified, current drinker, ex-drinker and non-drinker), body mass index (BMI) [overweight (BMI > 18.5 kg/m^2^), obesity (BMI ≥ 30.00 kg/m^2^) and underweight (BMI < 18.5 kg/m^2^)] and smoking status (current smoker, ex-smoker and non-smoker), as shown in Table 1.

The independent variables, which were the non-modifiable risk factors, comprised age in years (above 65, 55–64, 45–54, 35–44, 25–34, 15–24 and below 15), gender (female and male), educational level (unclassified, no formal, primary, secondary and tertiary), household income in Malaysian ringgit (RM) (above RM7,000, RM5,001–RM7,000, RM3,001–RM5,000, RM1,501–RM3,000, RM0–RM1,500), marital status (widow/widower/divorced, married and single), residential area (urban and rural), race (others, other Bumiputra, Indian, Chinese and Malays) and occupation (retired, homemaker, self-employed, private and government/semi government).

### Statistical Model

Multinomial logistic regression (MLR) is used to model the different possible outcomes (dependent variables) to predict the outcomes by estimating the odds of the modifiable and non-modifiable risk factors (polytomous variables) in this study. One category of dependent variables will be selected as the reference category when using this multinomial logistic regression. The reference category will be omitted when the odds of an event occurring in the presence of a factor, compared to the odds of an event occurring in the absence of that factor are determined for all independent variables for each category of the dependent variables. This is to establish multinomial logistic regression model by developing the relationship with the predictor variables for the purpose to estimate and assess the prediction of independent variables on the dependent variable. In multinomial logit model, data over the individual are analysed, the effects of the explanatory variables were allowed to differ for each outcome (6, 7). One of the advantages to apply multinomial logistic regression is Multinomial Logistic Regression (MLR) does not assume normality, linearity, or The specification for Multinomial Logit Model will be as follows:

When the outcomes of a response variable are polytomous with *k* nominal categories 2, *k* > 2, let *y* be the dependent variable with *k* nominal outcomes. The *k* categories are numbered 1 through *k*, but are not in ordered in any way. Let Pr (*y_i_*=*j*| *X_i_*) be the probability of observing outcome *j* for individual *i* given *X*, the set of explanatory variables. As a probability model, the Multinomial Logit Model can be written as:

$$\text{ln }\Omega_{j/r}(X)=\text{ln}\frac{\text{Pr}\;(y=j|X)}{\text{Pr}\;(y=r|X)}=X\beta_{j/r}\;for\;j=1\;to\;k$$

where *r* is the reference category, also referred to as the comparison group. These *k* equations can be solved to compute the predicted probabilities:

(1) $$\text{Pr}\;(y=j|X)=\frac{\text{exp}(X\beta_{j/r})}{\sum_{k=1}^{K}\text{exp}(X\beta_{k/r})}$$

The overall fit of the model has been assessed by using the Chi-square goodness-of-fit test estimation. Besides, the Multinomial Logit Model has been checked for possible multicollinearity by checking the variance inflation factor (VIF). Next, the level of significance of all tests is based on *P*-value of less than 5% (two-sided). The statistical analyses were done using the Statistical Package for Social Science (SPSS, Inc., Chicago, IL; version, 23.00).

## Results

### Characteristics of Respondents

Table 2 shows the demographic differences among the 24,632 eligible respondents. In total, 9,376 out of the 24,632 eligible respondents are hypertensive, and 4,537 out of the 9,376 (48.4%) hypertensive patients are found to be ‘known’ cases. By contrast, more 4,839 out of the 9,376 (51.6%) hypertensive patients are found to be undiagnosed with hypertension.

Table 3 presents the demographic factors of the sample consisting of gender, age, race, educational level, occupation, household income, residential area and marital status. In terms of gender, 2,434 (9.9%) males and 2,405 (9.8%) females suffer from undiagnosed hypertension. Known hypertensive patients comprise 2,484 (10.1%) females and 2,053 (8.3%) males. Majority of the secondary educated (7.5%) individuals show the highest percentage of having undiagnosed hypertension, and primary educated individuals (8.1%) have the highest percentage of being known hypertensive patients.

About 5.5% of the private sector employees show a high percentage of being undiagnosed hypertensive individuals, and homemakers (4.8%) account for a high percentage of being known hypertensive individuals. Low-income earners (RM0–RM1,500) show the highest percentage of being undiagnosed (7.2%) and known hypertensive (6.8%) individuals. Both urban (10.6%) and rural (10.1%) dwellers exhibit a high percentage of being undiagnosed and known hypertensive individuals. Overall, 11.7% of Malays are identified as undiagnosed hypertensive patients, and 10.9% of them are known hypertensive individuals. Married couples show a high percentage of being undiagnosed (11.8%) and known hypertensive individuals (8.9%). The respondents aged 45–54 years have the highest percentage of being undiagnosed hypertensive, and those aged 0–15 years have the highest percentage of being known hypertensive individuals.

### Diagnostic Tests for the Multinomial Logistic Model

Two tests of the null hypothesis show that the model adequately fits the data. The Pearson value is 0.303, which is smaller than 0.5 (50%), and the deviance value is 1.000, which is also smaller than 0.5 (50%). The Pearson value of 0.303 indicates that the data are not consistent with the model assumptions. The checking of multicollinearity reveals that no collinearity exists among the independent variables as the variance inflation factor is less than 10. In the cases used to create the model, 14,408 of the 21,422 people who are healthy (no hypertension) are classified correctly; 335 of the 4,834 who suffered from undiagnosed hypertension are classified correctly; and 1,025 of the 4,523 who suffered from known hypertension are classified correctly. Overall, 64.1% of the cases are classified correctly.

### Results for the Modifiable Risk Factors in Undiagnosed and Known Hypertension

The results from the estimated multinomial logistic regression are presented in Table 4. The first portion of the regression compares the respondents who have undiagnosed hypertension with those who have no hypertension. The second part of the regression compares those who have known hypertension with those who have no hypertension. The results show that physical activity significantly (*P* < 0.001) affects the chances of being diagnosed with undiagnosed hypertension due to physical inactivity. The physically inactive respondents are 0.862 times more likely to suffer from undiagnosed hypertension than the physically active respondents. Not all drinking status affects the chances of having undiagnosed hypertension. Only current drinkers significantly (*P* = 0.003) show higher odds (OR = 1.225) of having undiagnosed hypertension than nondrinkers. BMI is a significant (*P* < 0.001) variable affecting the probability of having undiagnosed hypertension. The odds of having undiagnosed hypertension for obese and overweight respondents are 2.112 and 1.513, respectively, in comparison with normal-weight respondents.

In the second part of the regression, the odds of having known hypertension is 1.154 times higher (with *P* < 0.001) in those who are physically inactive than in the reference category of physically active respondents. The unclassified drinkers have a significantly (*P* < 0.001) higher likelihood of having known hypertension (OR = 2.370; 95% CI: 1.717, 3.271) than the nondrinkers. However, current drinkers have the odds of known hypertension that are significantly (*P* = 0.029) less than 1 (OR = 0.836), indicating that the current drinkers are less likely to have known hypertension. Smoking status is positively related to the likelihood of having known hypertension. The results show that ex-smokers have a higher likelihood (OR = 1.220) of suffering from known hypertension than non-smokers. Conversely, current smokers have significantly (*P* < 0.001) decreased odds (OR = 0.817) of having known hypertension. The odds of having known hypertension among those who are obese and overweight are 2.608 and 1.847, respectively, in comparison with the normal-weight respondents.

### Results for the Non-Modifiable Risk Factors in Undiagnosed and Known Hypertension

The results also indicate that the females have a considerably (*P* < 0.001) lesser likelihood (OR = 0.848; 95% CI: 0.785, 0.916) of having undiagnosed hypertension than the males. In the case of race, the odds ratio for Chinese and Indians are less than 1 (0.824 and 0.826 respectively), suggesting that the Chinese and Indian respondents are less likely to have undiagnosed hypertension than Malays. Age also significantly (*P* < 0.001) affects the likelihood of having undiagnosed hypertension among the respondents. The OR in favour of having undiagnosed hypertension for the respondents aged over 65, 55–64, 45–54 and 35–44 years are 7.518, 5.264, 3.205 and 2.125, respectively, as compared to the respondents aged below 15 years. The respondents aged 15–24 years have an OR of having undiagnosed hypertension of significantly (*P* < 0.001) less than 1 (OR = 0.695), suggesting that those who are at a lower age group are less likely to be diagnosed with undiagnosed hypertension.

Educational level is a significant (*P* < 0.001) variable affecting the chances of having undiagnosed hypertension. The OR for the respondents with no formal education, primary education and secondary education are greater than 1 (1.426, 1.475 and 1.310, respectively), indicating that those with less education are also more likely to suffer from undiagnosed hypertension. The OR of undiagnosed hypertension is significantly (*P* < 0.001) lower among the urban residents (OR = 0.924; 95% CI: 0.859, 0.994) than among rural dwellers. Household income significantly (*P* = 0.002, *P* = 0.047) affects the likelihood of having undiagnosed hypertension among the respondents. The OR for the respondents with a household income above RM7,000 and RM5,001–RM7,000 are less than 1 (0.806 and 0.871 respectively), suggesting that those with a higher income level are less likely to suffer from undiagnosed hypertension. Married couples have significantly (*P* < 0.001) decreased odds (OR = 0.770; 95% CI: 0.674, 0.879) of undiagnosed hypertension compared with the single respondents. All smoking statuses, inadequate fruit and vegetable consumption, ex-drinkers, unclassified drinkers, underweight respondents, other ethnic respondents, unclassified education, all types of occupation and widow/widower are not associated with any likelihood of having undiagnosed hypertension among individuals in Malaysia.

This study shows that only other Bumiputras have a considerably (*P* = 0.013) higher probability (OR = 1.174; 95% CI: 1.034, 1.333) of having known hypertension than Malays. The odds ratio of suffering from known hypertension is 5.418, 3.417 and 1.501 times greater (with *P* < 0.001) among those over 65, 55–64 and 45–54 years of age, respectively, than the reference age category of below 15 years old. The respondents who are 35–44, 25–34 and 15–24 years old have an odds ratio of being known hypertensive of significantly (*P* < 0.001) less than 1 at 0.558, 0.197 and 0.496, respectively, suggesting that the younger age groups are less likely to be known hypertensive. Additionally, educational level has been found to significantly (*P* < 0.001) affect the likelihood of having known hypertension among individuals. For example, the respondents who have unclassified education or no formal education, primary education or secondary education are respectively 1.785, 1.786, 1.798 and 1.586 times likely to suffer from known hypertension than the respondents who have completed their tertiary education. Occupation is found to be a significant variable affecting the likelihood of having known hypertension. The odds ratio for respondents who are retirees, self-employed and private workers are less than 1 (0.829, 0.811 and 0.786, respectively), suggesting that private workers are less likely to suffer from known hypertension. Married couples are found to have a significantly (*P* = 0.020) lower likelihood of being known hypertensive (OR = 0.815) than singles. Fruit and vegetable consumption, residential area, household income and gender do not have a significant difference in the likelihood of having known hypertension among the respondents.

## Discussion

### Modifiable Risk Factors Affecting the Odds of Getting Undiagnosed and Known Hypertension

With regard to physical activity, this study shows that the physically inactive respondents are less likely to have undiagnosed hypertension. This result is inconsistent with that in the study of Zhang et al. (9), which revealed that physical inactivity was significantly associated with the increased odds of undiagnosed hypertension among urban Chinese adults. This contradiction may be due to the fact that the research was conducted with a distinct background in different countries. Other factors, for example, excess alcohol consumption, may have contributed to the higher odds of undiagnosed hypertension.

The present study shows that only current drinkers have significantly higher odds of having undiagnosed hypertension, consistent with the finding of Zhang et al. (9) that alcohol drinking was significantly associated with the increased odds of undiagnosed hypertension in China. Therefore, the government should conduct a health awareness campaign among current drinkers to monitor the prevalence of undiagnosed hypertension. Another finding of this study indicates a significant difference between the obese and overweight respondents and the likelihood of having undiagnosed hypertension. This result lends support to the work of Bushara et al. (5), who found that increased weight led to the increased prevalence of undiagnosed hypertension and that obese respondents had highest prevalence (46.5%) of undiagnosed hypertension.

Moreover, this study is consistent with that of Olack et al. (10), who found that individuals with a moderate level of physical activity had higher odds of suffering from known hypertension in Kenya. Therefore, government interventions on active lifestyle should be emphasised in the public and private sectors by promoting more sports activities among Malaysians. The findings of this study show that the current and unclassified drinkers are more likely to be diagnosed as known hypertensive. This result is inconsistent with that of Ibekwe (11), who reported a significant association between drinking (*P* < 0.001) and hypertension: the respondents who were hypertensive and consumed alcohol accounted for 33.1% (39/118), and those who did not consume alcohol were 66.9% (79/118). Moreover, frequent alcohol consumption was found to increase the probability of having hypertension in China (9).

Smoking is found to be significantly associated with the likelihood of having known hypertension in this study. Ex-smokers have significantly higher odds of having known hypertension than non-smokers, consistent with the previous research that found regular and long cigarette smoking to be associated with hypertension (10, 11). Kannan and Satyamoorthy (15) found that the prevalence of hypertension was higher (33.3%) among those who were in the habit of chewing tobacco for more than five years than the prevalence of hypertension (31.6%) of those who had this habit for less than five years. By contrast, the present study shows that current smokers exhibit a lower likelihood of having known hypertension. Therefore, promoting awareness through campaigns to stop smoking is important to prevent the likelihood of being diagnosed with known hypertension, as smoking-related diseases such as cancer and cardiovascular disease are the main causes of premature death globally (13).

This study shows that obese respondents have significantly higher odds of suffering from known hypertension than normal-weight respondents. This finding agrees with those of Forman et al. (17) and Rampal et al. (18), who reported that overweight and obesity, high sodium intake, physical inactivity, heavy alcohol intake, low potassium intake and a Western-style diet are the major modifiable risk factors for hypertension. Obesity was identified as a well-established risk factor for cardiovascular diseases in the general population by Rampal et al. (18). Flack et al. (19) reported that obesity is linked to increased blood pressure, salt sensitivity, glucose intolerance and dyslipidaemia. Overweight and obese participants were found to be approximately 2.0 times more likely to be hypertensive than their counterparts with a normal BMI (17). In one study, the risks for hypertension in subjects with a BMI above 27 kg/m^2^ greatly increased, and these particular subjects had a two-fold higher relative risk than those with a BMI of less than 18.5 kg/m^2^ (18). Therefore, educational programmes to instil awareness of maintaining a healthy weight based on the BMI guidelines from the Ministry of Health are important to prevent and monitor the occurrence of known hypertension among Malaysians.

### Non-Modifiable Risk Factors Affecting the Odds of Getting Undiagnosed and Known Hypertension

The female respondents have a statistically significant lower likelihood of being undiagnosed hypertensive. This result is inconsistent with that of Cuschieri et al. (22), who found that females have a prevalence of undiagnosed hypertension of 39.3%, which is slightly higher than that of males at 36.7%). However, among Chinese urban adults, the male respondents were found to have increased odds of having undiagnosed hypertension (6). Similarly, Maltee males tended to be more likely (64.01%; 95% CI 95%: 58.56, 69.13) to have undiagnosed hypertension as reported by Cuschieri et al. (22). The results of the current study show that both Indians and Chinese have low odds of being diagnosed with undiagnosed hypertension.

In terms of age, this study demonstrates that the higher age groups have significantly higher odds of having undiagnosed hypertension. This result is consistent with that of Bushara et al. (5), who found the highest prevalence in the participants above 65 years and therefore a significant association between undiagnosed hypertension and increasing age (*P* < 0.05). The result is also in agreement with that of Zhang et al. (9), who found older age to be associated with higher odds of undiagnosed hypertension in China. In terms of education, the current study shows that the respondents with a lower educational level have significantly higher odds of having undiagnosed hypertension. This result is consistent with that of El Fadil et al. (23), who reported that lower educational status and illiteracy led to a higher prevalence (34.9%) of undiagnosed hypertension.

This study also reveals that the likelihood of having undiagnosed hypertension decreases among the urban residents (OR = 0.924) in comparison with the other rural dwellers. Consequently, the rural respondents are shown to be more likely to have undiagnosed hypertension. This finding is inconsistent with that of Hou (12), who found the undiagnosed hypertension rate to be significantly higher in the countryside than in the city. The probable reason for this result could be that the rural population was older, which led to the higher likelihood of having undiagnosed hypertension as reported by Cheah et al. (24). Interventions targeting rural adults should promote awareness of hypertension among Malaysians.

The findings demonstrate that the higher income group has significantly lower odds of having undiagnosed hypertension. This outcome lends support to the study of Zhang et al. (9), which also reported that higher income earners have lower odds of having undiagnosed hypertension. Higher income earners are suggested to have better access to medical facilities, for example, health screening to monitor blood pressure, and can thus prevent the likelihood of having undiagnosed hypertension.

The married couples tend to have statistically significant lower odds of having undiagnosed hypertension than the single respondents. This outcome is consistent with that of Mosca and Kenny (25), who found that married adults were less likely to have high blood pressure objectively in the United States but not in Ireland. In a previous study, married individuals were found to have potentially greater financial resources available for health care and for promoting a healthier lifestyle (23). Occupation does not show any significant difference in the likelihood of having undiagnosed hypertension among Malaysians.

Socio-demographic factors play an important role as determinants of the daily activities of individuals. The findings demonstrate that age, educational level, marital status, gender, residential area, race, household income and occupation are statistically significant in regulating individuals’ chances of having various hypertensive outcome levels. This study shows that gender is not significantly associated with the likelihood of having known hypertension among Malaysians, inconsistent with the findings of Azimi-Nezhad (27). The results of this study are comparable with those of a previous study indicating no significant difference in gender in the likelihood of having known hypertension (25). Other factors or predictors may contribute to the likelihood of having known hypertension among individuals.

This study shows that only other Bumiputras have significantly higher odds of having known hypertension and this finding is by supported by Omar et al.’s (29) study, which reported that other Bumiputras were 1.55 times more likely to have hypertension than Malays. This disparity may be due to the differences in genetic or socio-environmental factors. Moreover, the findings of this study are consistent with those reported by Cheah et al. (24), El Fadil et al. (23) and Gao et al. (30), who revealed that a higher age group is more likely to suffer from known hypertension. Gao et al. (30) reported that the prevalence of hypertension increases in relation to a higher age group at 13.0%, 36.7% and 56.5% among the respondents aged 20–44 years (young people), 45–64 years (middle-aged people) and equal to or greater than 65 years (elderly people), respectively (27). The findings of this study also closely follow those of Bushara et al. (5), who identified a lower educational level to be more likely to increase the risk of having known hypertension. However, this study does not show any significant difference in residential area, household income level and the likelihood of being known hypertensive patients. The results reveal that retirees, self-employed and private sector respondents are less likely to have known hypertension, consistent with the finding of Hou (12), who found retirees to have a statistically significant lower likelihood of suffering from known hypertension as retirement seemed to be beneficial for lowering blood pressure in the Chinese context.

Mosca and Kenny (25) found that married adults were less likely to have high blood pressure. This result supports the finding of this research, which shows that married couples have statistically significant lower odds of having known hypertension than the single respondents. The probable reason for this outcome may be that married couples bear greater responsibility in taking care of their families and are thus more aware in terms of monitoring their blood pressure and health status through health screening programmes. Therefore, this study provides valuable information to relevant authorities and helps in the implementation of government intervention programmes to focus on and control the prevalence of undiagnosed and known hypertension among singles and the older age group.

## Conclusion

The present study finds that among the modifiable risk factors, the significant predictors of obese and overweight individuals and current drinkers have higher odds of being undiagnosed hypertension patients. Physically inactive respondents exhibit lower odds of being undiagnosed hypertension patients. Moreover, older respondents (above 65 years old) and respondents with primary education are shown to have higher odds of having undiagnosed hypertension. Female respondents, the youngest age group (15–24 years old), urban dwellers, Chinese and Indian, high income earners and retirees are found to have statistically significant lower odds of having undiagnosed hypertension.

Among the modifiable risk factors, the significant predictors (i.e., the respondents who are obese and overweight, physically inactive, unclassified drinkers, ex-smokers) increase their chances of having known hypertension. Current smokers show lower odds of being known hypertension patients. Other significant predictors such as older respondents (above 65 years old), other Bumiputras and primary educated respondents show higher odds of having known hypertension. Conversely, retirees, self-employed, private employees and married couples have significantly lower odds of being diagnosed with known hypertension.

This study has some limitations. Firstly, using secondary data is challenging because identifying the details, especially the definition of the variables of the secondary data, is necessary. Secondly, this study is limited by its cross-sectional nature, which did not allow us to make any conclusive statement about the temporality of the observed associations.

## Figures and Tables

**Table 1:** Categorical variable coding for modifiable risk factors

Modifiable Risk Factor(s)	Variable Coding(s)	Definition
Physical activity	1=Inactive	There is no activity is reported or some activity is reported but not enough to meet moderate or high categories
	2=Active (Reference)	If his/ her combination of vigorous-intensity, moderate-intensity and walking activities achieved a minimum 0f 600 MET-minutes per week
Drinking status (define and analysis based on respondent’s answer)	0=Unclassified	Declared as current drinker in question B9100 but did not answered module L
	1=Current drinker	Respondent who is still consuming alcoholic beverages for the past 12 months
	2=Ex-drinker	The respondent was previously a drinker
	3=Non-drinker (Reference)	The respondent is a non-drinker
Smoking status	0=Current smoker	The respondent is a current smoker
	1=Ex-smoker	The respondent was previously a smoker
	2=Non-smoker (Reference)	The respondent is a non-smoker
Fruit and vegetables consumption (based on STEPS WHO criteria)	1=Inadequate	< 5 servings per day
	0=Adequate (Reference)	≥ 5 servings per day
Body Mass Index (BMI) status (WHO1998)	0=Obese	≥ 30.0 kg/m^2^
	1=Overweight	25.0–29.99 kg/m^2^
	2=Underweight	< 18.5 kg/m^2^
	3=Normal weight (Reference)	18.5–24.99 kg/m^2^

**Table 2:** Frequency of hypertension (HP)

Hypertension	Frequency	Percent
No HP	15,256	61.9
Newly HP	4,839	19.6
Known HP	4,537	18.4
Total	24,632	100.0

**Table 3:** Demographic and socioeconomic characteristics of respondents with different hypertension outcome levels

Variable(s)	Level(s)	No hypertension Frequency (%)	Undiagnosed hypertension Frequency (%)	Known hypertension Frequency (%)
**Gender**	Male	7,317 (29.7)	2,434 (9.9)	2,053 (8.3)
	Female	7,939 (32.2)	2,405 (9.8)	2,484 (10.1)
**Education level**	Unclassified	1,026 (4.2)	227 (0.9)	408 (1.7)
	No formal education	622 (2.5)	443 (1.8)	494 (2.0)
	Primary education	4,274 (17.4)	1,781 (7.2)	2,000 (8.1)
	Secondary education	6,328 (25.7)	1,838 (7.5)	1,318 (5.4)
	Tertiary education	3,006 (12.2)	550 (2.2)	317 (1.3)
**Occupation**	Retire	1,588 (6.4)	790 (3.2)	992 (4.0)
	Home maker	2,963 (12.0)	1,039 (4.2)	1,178 (4.8)
	Self employed	3,058 (12.4)	1,143 (4.6)	909 (3.7)
	Private	5,574 (22.6)	1,348 (5.5)	936 (3.8)
	Government/Semi-government	2,073 (8.4)	519 (2.1)	522 (2.1)
**Household income**	Above RM7,000	1,775 (7.2)	406 (1.6)	442 (1.8)
	RM5,001–RM7,000	1,458 (5.9)	370 (1.5)	386 (1.6)
	RM3,001–RM5,000	3,239 (13.1)	935 (3.8)	838 (3.4)
	RM1,501–RM3,000	4,157 (16.9)	1,355 (5.5)	1,195 (4.9)
	RM0–RM1,500	4,627 (18.8)	1,773 (7.2)	1,676 (6.8)
**Residential area**	Urban	9,076 (36.8)	2,612 (10.6)	2,492 (10.1)
	Rural	6,180 (25.1)	2,227 (9.0)	2,045 (8.3)
**Race**	Others	951 (3.9)	239 (1.0)	144 (0.6)
	Other Bumiputra	1,463 (5.9)	515 (2.1)	463 (1.9)
	Indian	1,188 (4.8)	353 (1.4)	365 (1.5)
	Chinese	2,734 (11.1)	861 (3.5)	891 (3.6)
	Malays	8,920 (36.2)	2,871 (11.7)	2,674 (10.9)
**Marital status**	Widow/widower or Divorced	519 (2.1)	446 (1.8)	505 (2.1)
	Married	7,443 (30.2)	2,915 (11.8)	2,179 (8.9)
	Single	7,281 (29.6)	1,475 (6.0)	1,842 (7.5)
**Age**	> 65 years old	474 (1.9)	573 (2.3)	720 (2.9)
	55–64 years old	866 (3.5)	767 (3.1)	827 (3.4)
	45–54 years old	1,802 (7.3)	943 (3.8)	737 (3.0)
	35–44 years old	2,465 (10.0)	829 (3.4)	348 (1.4)
	25–34 years old	3,279 (13.3)	551 (2.2)	156 (0.6)
	15–24 years old	3,571 (14.5)	469 (1.9)	517 (2.1)
	< 15 years old	2,799 (11.4)	707 (2.9)	1,232 (5.0)

**Table 4:** Parameter estimates for multinomial logistic regressions on hypertension

Hypertension	Predictor(s)	B Coefficient	Std. Error	Exp (B) Odds Ratio	95% Confidence Interval for Exp (B)		*P*-value
					Lower bound	Upper bound	
Undiagnosed Hypertension	Intercept	2.007	0.126		< 0.001	< 0.001	< 0.001

	**Age**						
	> 65 years old	2.017	0.114	7.518	< 0.001	< 0.001	< 0.001
	55–64 years old	1.661	0.098	5.264	< 0.001	< 0.001	< 0.001
	45–54 years old	1.165	0.095	3.205	< 0.001	< 0.001	< 0.001
	35–44 years old	0.754	0.096	2.125	0.368	0.368	0.368
	25–34 years old	0.081	0.090	1.085	< 0.001	< 0.001	< 0.001
	15–24 years old	0.363	0.078	0.695	–	–	–
	below 15 yrs old (R)	0^b^	–	–			
	**Marital status**						
	Widow/widower or divorced	0.074	0.101	0.928	0.463	0.463	0.463
	Married	0.262	0.068	0.770	< 0.001	< 0.001	< 0.001
	Single (R)	0^b^	–	–	–	–	–
	**Gender**						
	Female	0.165	0.039	0.848	< 0.001	< 0.001	< 0.001
	Male (R)	0^b^	–	–	–	–	–
	**Physical activity**						
	Inactive	0.149	0.038	0.862	< 0.001	< 0.001	< 0.001
	Active (R)	0^b^	–	–	–	–	–
	**Residential are**a						
	Urban	0.079	0.037	0.924	0.034	0.034	0.034
	Rural (R)	0^b^	–	–	–	–	–
	**Race**						
	Others	0.055	0.082	0.946	0.806	1.111	0.499
	Other Bumiputra	0.101	0.061	1.106	0.981	1.247	0.099
	Indian	0.191	0.070	0.826	0.721	0.946	0.006
	Chinese	0.194	0.052	0.824	0.743	0.912	< 0.001
	Malays (R)	0^b^	–	–	–	–	–
	**Occupatio**n						
	Retire	0.085	0.078	1.089	0.935	1.268	0.273
	Home maker	0.131	0.068	1.139	0.997	1.302	0.055
	Self-employed	0.073	0.066	1.076	0.946	1.224	0.267
	Private	0.055	0.063	1.057	0.935	1.195	0.375
	Gov/Semi-Gov (R)	0^b^	–	–	–	–	–
	**Household inco**me						
	Above RM7,000	0.216	0.068	0.806	0.705	0.922	0.002
	RM5,001–RM7,000	0.138	0.070	0.871	0.760	0.998	0.047
	RM3,001–RM5,000	0.064	0.051	0.938	0.849	1.036	0.207
	RM1,501–RM3,000	0.000	0.045	1	0.916	1.093	0.996
	RM0–RM1,500 (R)	0^b^	–	–	–	–	–
	**Fruit & vege consumption**						
	Inadequate	0.019	0.068	1.020	0.893	1.164	0.773
	Adequate (R)	0^b^	–	–	–	–	–
	**Drinking status**						
	Unclassified	0.267	0.205	1.307	0.875	1.952	0.191
	Current drinker	0.203	0.067	1.225	1.074	1.398	0.003
	Ex-drinker	0.069	0.085	0.933	0.789	1.103	0.417
	Non-drinker (R)	0^b^	–	–	–	–	–
	**Smoking statu**s						
	Current smoker	0.026	0.047	0.974	0.889	1.068	0.578
	Ex-smoker	0.145	0.085	1.156	0.978	1.366	0.089
	Non-smoker (R)	0^b^	–	–	–	–	–
	**Education leve**l						
	Unclassified	0.190	0.103	1.209	0.988	1.479	0.065
	No formal	0.355	0.095	1.426	1.183	1.718	< 0.001
	Primary	0.389	0.069	1.475	1.289	1.688	< 0.001
	Secondary	0.270	0.059	1.310	1.167	1.469	< 0.001
	Tertiary (R)	0^b^	–	–	–	–	–
	**Body Mass Index**						
	Obese	0.841	0.048	2.318	2.112	2.545	< 0.001
	Overweight	0.496	0.042	1.642	1.513	1.783	< 0.001
	Underweight	0.106	0.076	0.899	0.775	1.043	0.161
	Normal weight (R)	0^b^	–	–	–	–	–
Known Hypertension	Intercept	−1.883	0.135				< 0.001

	**Age**						
	> 65 years old	1.690	0.123	5.418	4.255	6.898	< 0.001
	55–64 years old	1.229	0.109	3.417	2.759	4.231	< 0.001
	45–54 years old	0.406	0.108	1.501	1.214	1.856	< 0.001
	35–44 years old	−0.583	0.115	0.558	0.445	0.699	< 0.001
	25–34 years old	−1.625	0.119	0.197	0.156	0.249	< 0.001
	15–24 years old	−0.702	0.074	0.496	0.429	0.573	< 0.001
	below 15 yrs (R)	0^b^	–	–	–	–	–
	**Marital status**						
	Widow/widower or divorced	−0.030	0.116	0.970	0.772	1.219	0.794
	Married	−0.205	0.088	0.815	0.686	0.968	0.020
	Single (R)	0^b^	–	–	–	–	–
	**Gender**						
	Female	−0.004	0.041	0.996	0.919	1.078	0.914
	Male (R)	0^b^	–	–	–	–	–
	**Physical activity**						
	Inactive	0.143	0.038	1.154	1.070	1.244	< 0.001
	Active (R)	0^b^	–	–	–	–	–
	**Residential area**						
	Urban	0.064	0.039	0.938	0.869	1.014	0.106
	Rural (R)	0b	–	–	–	–	–
	**Race**						
	Others	0.161	0.100	0.851	0.700	1.036	0.108
	Other Bumiputra	0.160	0.065	1.174	1.034	1.333	0.013
	Indian	0.007	0.071	1.007	0.877	1.157	0.918
	Chinese	0.030	0.053	0.970	0.875	1.075	0.562
	Malays (R)	0^b^	–	–	–	–	–
	**Occupation**						
	Retire	0.187	0.075	0.829	0.716	0.960	0.012
	Home maker	0.043	0.068	1.044	0.914	1.193	0.524
	Self-employed	0.209	0.069	0.811	0.708	0.929	0.003
	Private	0.241	0.067	0.786	0.690	0.896	< 0.001
	Gov/Semi gov (R)	0^b^	–	–	–	–	–
	**Household income**						
	Above RM7,000	0.055	0.069	1.056	0.923	1.210	0.427
	RM5,001–RM7,000	0.089	0.071	1.093	0.951	1.257	0.210
	RM3,001–RM5,000	0.014	0.054	1.014	0.913	1.127	0.793
	RM1,501–RM3,000	0.022	0.048	1.022	0.931	1.123	0.642
	RM0–RM1,500 (R)	0^b^	–	–	–	–	–
	**Fruit & vegetables consumption**						
	Inadequate	0.127	0.075	1.136	0.980	1.316	0.091
	Adequate (R)	0^b^	–	–	–	–	–
	**Drinking status**						
	Unclassified	0.863	0.164	2.370	1.717	3.271	< 0.001
	Current drinker	0.179	0.082	0.836	0.712	0.981	0.029
	Ex-drinker	0.088	0.089	1.092	0.917	1.299	0.325
	Non-drinker (R)	0^b^	–	–	–	–	–
	**Smoking status**						
	Current smoker	0.202	0.046	0.817	0.746	0.895	< 0.001
	Ex-smoker	0.199	0.089	1.220	1.025	1.452	0.025
	Non-smoker (R)	0^b^	–	–	–	–	–
	**Education level**						
	Unclassified	0.580	0.102	1.785	1.785	2.181	< 0.001
	No formal	0.580	0.105	1.786	1.786	2.195	< 0.001
	Primary	0.587	0.081	1.798	1.798	2.108	< 0.001
	Secondary	0.461	0.073	1.586	1.586	1.829	< 0.001
	Tertiary (R)	0^b^	–	–	–	–	–
	**Body Mass Index**						
	Obese	0.959	0.050	2.608	2.364	2.877	< 0.001
	Overweight	0.614	0.046	1.847	1.690	2.020	< 0.001
	Underweight	0.006	0.078	0.994	0.853	1.159	0.942
	Normal weight (R)	0^b^	–	–	–	–	–

## References

[1] Organization WH (2012). Malaysia health system review.

[2] Kearney PM, Whelton M, Reynolds K, Muntner P, Whelton PK, He J (2005). Global burden of hypertension: analysis of worldwide data. The Lancet.

[3] Sameerah S, Sarojini S, Goh A, Faridah A, Rosminah M, Radzi H (2007). Malaysian statistics on medicine 2005.

[4] Health IfP (2011). National Health and Morbidity Survey 2011 (NHMS 2011).

[5] Bushara SO, Noor SK, Elmadhoun WM, Sulaiman AA, Ahmed MH (2015). Undiagnosed hypertension in a rural community in Sudan and association with some features of the metabolic syndrome: how serious is the situation?. Ren Fail.

[6] Long JS (1997). Advanced quantitative techniques in the social sciences series, Vol. 7. Regression models for categorical and limited dependent variables.

[7] Porter SR (1999). Viewing one-year retention as a continuum: the use of dichotomous logistic regression, ordered logit and multinomial logit.

[8] Starkweather J, Moske AK (2011). Multinomial logistic regression. http://www.unt.edu/rss/class/Jon/Benchmarks/MLR_JDS_Aug2011.pdf.

[9] Zhang H, Deng M, Xu H, Wang H, Song F, Bao C (2017). Pre-and undiagnosed-hypertension in urban Chinese adults: a population-based cross-sectional study. J Hum Hypertens.

[10] Olack B, Wabwire-Mangen F, Smeeth L, Montgomery JM, Kiwanuka N, Breiman RF (2015). Risk factors of hypertension among adults aged 35–64 years living in an urban slum Nairobi, Kenya. BMC Public Health.

[11] Ibekwe R (2015). Modifiable risk factors of hypertension and socio demographic profile in Oghara, Delta state; prevalence and correlates. Ann Med Health Sci Res.

[12] Hou X (2008). Urban—rural disparity of overweight, hypertension, undiagnosed hypertension, and untreated hypertension in China. Asia Pac J Public Health.

[13] Abdulsalam S, Olugbenga-Bello A, Olarewaju O, Abdus-Salam I (2014). Sociodemographic correlates of modifiable risk factors for hypertension in a rural local government area of oyo state south west Nigeria. Int J Hypertens.

[14] Alikor CA, Emem-Chioma PC, Odia OJ (2013). Hypertension in a rural community in Rivers State, Niger Delta region of Nigeria: prevalence and risk factors. Nigerian Health Journal.

[15] Kannan L, Satyamoorthy T (2009). An epidemiological study of hypertension in a rural household community. Sri Ramachandra J Med.

[16] Bonita R, Magnusson R, Bovet P, Zhao D, Malta DC, Geneau R (2013). Country actions to meet UN commitments on non-communicable diseases: a stepwise approach. The Lancet.

[17] Forman JP, Stampfer MJ, Curhan GC (2009). Diet and lifestyle risk factors associated with incident hypertension in women. JAMA.

[18] Rampal L, Rampal S, Khor GL, Zain AM, Ooyub SB, Rahmat RB (2007). A national study on the prevalence of obesity among 16,127 Malaysians. Asia Pac J Clin Nutr.

[19] Flack JM, Ferdinand KC, Nasser SA (2003). Epidemiology of hypertension and cardiovascular disease in African Americans. J Clin Hypertens.

[20] Mbochi RW, Kuria E, Kimiywe J, Ochola S, Steyn NP (2012). Predictors of overweight and obesity in adult women in Nairobi Province, Kenya. BMC Public Health.

[21] Ishikawa-Takata K, Ohta T, Moritaki K, Gotou T, Inoue S (2002). Obesity, weight change and risks for hypertension, diabetes and hypercholesterolemia in Japanese men. Eur J Clin Nutr.

[22] Cuschieri S, Vassallo J, Calleja N, Pace N, Mamo J (2017). The effects of socioeconomic determinants on hypertension in a cardiometabolic at-risk European country. Int J Hypertens.

[23] El Fadil MO, Suleiman I, Alzubair AG (2007). Clinico-epidemiological features of hypertensive subjects in kassala town, Eastern Sudan. J Family Community Med.

[24] Cheah WL, Lee PY, Khatijah Y, Rasidah AW (2011). A preliminary study on the prevalence of cardiovascular disease risk factors in selected rural communities in samarahan and kuching division, sarawak, malaysia. Malays J Med Sci.

[25] Mosca I, Kenny RA (2014). Exploring differences in prevalence of diagnosed, measured and undiagnosed hypertension: the case of Ireland and the United States of America. Int J Public Health.

[26] Lipowicz A, Lopuszanska M (2005). Marital differences in blood pressure and the risk of hypertension among Polish men. Eur J Epidemiol.

[27] Azimi-Nezhad M, Ghayour-Mobarhan M, Parizadeh M, Safarian M, Esmaeili H, Parizadeh S (2008). Prevalence of type 2 diabetes mellitus in Iran and its relationship with gender, urbanisation, educatio, marital status and occupation. Singapore Med J.

[28] Bharati D, Nandi P, Yamuna T, Lokeshmaran A, Agarwal L, Singh J (2012). Prevalence and covariates of undiagnosed hypertension in the adult population of Puducherry, South India. Nepal Journal of Epidemiology.

[29] Omar MA, Irfan NI, Yi KY, Muksan N, Majid NLA, Yusoff MFM (2016). Prevalence of young adult hypertension in Malaysia and its associated factors: findings from National Health and Morbidity Survey 2011. Malaysian Journal of Public Health Medicine.

[30] Gao Y, Chen G, Tian H, Lin L, Lu J, Weng J (2013). Prevalence of hypertension in China: a cross-sectional study. PloS One.

